# The application of myocardial contrast echocardiography in assessing microcirculation perfusion in patients with acute myocardial infarction after PCI

**DOI:** 10.1186/s12872-021-02404-9

**Published:** 2022-05-20

**Authors:** Wei-yang Lyu, Chuan-yu Qin, Xiao-tong Wang, Sheng-long Shi, Hui-lin Liu, Jia-wei Wang

**Affiliations:** 1 Department of Ultrasound Medicine, The Second Affiliated Hospital of Qiqihar Medical University, 37 Zhonghua West Road, Jianhua District, Qiqihar, 161006 China; 2 Department of Radiology, Tongjiang People’s Hospital, Tongjiang City, 156400 China

**Keywords:** ST-segment elevation myocardial infarction, Percutaneous coronary intervention, Microcirculatory resistance, Myocardial contrast echocardiography, Myocardial function

## Abstract

**Background:**

To evaluate the myocardial microcirculation perfusion of patients with acute ST-segment elevation myocardial infarction (STEMI) with a different index of microcirculatory resistance (IMR) after percutaneous coronary intervention (PCI) by myocardial contrast echocardiography (MCE) and analyse the value of MCE in predicting myocardial perfusion after PCI.

**Methods:**

Fifty-six patients with acute STEMI who underwent an emergency PCI were selected from October 2018 to October 2019 in our hospital. According to the IMR values measured during PCI treatment, the patients were divided into three groups. Traditional ultrasound and MCE were performed one week after PCI. The left ventricular ejection fraction (LVEF), ventricular wall motion score index (WMSI), A value, β value and A × β value (which refers to the patient’s myocardial blood flow) were measured. The receiver operating characteristic curve was drawn to evaluate the effectiveness of the MCE parameters in the diagnosis of myocardial microcirculation perfusion disorders.

**Results:**

The results showed that there was no significant difference in the LVEF among the groups. The WMSI in Group 3 was statistically different from that in Groups 1 and 2 (*P* < 0.05), but there was no statistically significant difference in the WMSI between Groups 1 and 2. Among the three groups, the A value, β value and A × β value were significantly different (*P* < 0.05). According to Spearman’s correlation analysis, the MCE quantitative parameters (i.e. the A value, β value and A × β value) were negatively correlated with the IMR value (r = −0.523, −0.471, −0.577, *P* < 0.01).

**Conclusions:**

The A value, β value and A × β value were negatively correlated with the IMR value. Furthermore, MCE could be used to observe the myocardial perfusion in patients with acute STEMI after PCI and may be one of the indicators used to accurately evaluate myocardial microcirculation.

## Background

Percutaneous coronary intervention (PCI) is the most effective treatment for early coronary revascularisation in patients with acute coronary syndrome, especially for patients with acute ST-segment elevation myocardial infarction (STEMI). However, 10–40% of patients still experience adverse effects, such as unrelieved clinical symptoms and unimproved cardiac function after PCI [1]. This is because the myocardial tissue is still hypoperfused. Although the infarct-related epicardial coronary occlusion is relieved, this results in failure to complete the effective reperfusion of the myocardial tissue, which triggers a condition of low or no reflow and adversely affects the prognosis of patients [2]. Therefore, effective evaluation of myocardial perfusion after PCI in patients with acute STEMI has important clinical significance. The index of microcirculatory resistance (IMR), which is not affected by epicardial hemodynamic status and has the advantages of reliability and reproducibility, is an essential method to assess coronary microcirculatory function and is currently considered the most accurate indicator for evaluating microcirculation [3]. However, clinical application of IMR is limited because it is invasive and expensive. Myocardial contrast echocardiography (MCE) can be used to quantitatively assess myocardial microcirculatory perfusion in patients with coronary heart disease after PCI at the microcirculatory level using ultrasound contrast agents to visualise myocardial perfusion while observing left ventricular wall motion. It has the advantages of safety, non-invasiveness and convenience [4].

This study intended to detect the IMR and MCE in patients with acute STEMI who were treated with PCI and analyse the correlations to investigate the clinical value of MCE in evaluating myocardial microcirculatory perfusion after PCI.

## Methods

### Subjects

In this study, patients with acute STEMI for the first time admitted to our hospital from November 2018 to November 2019 were the main study population. All STEMI patients within 12 h of onset were treated with PCI surgery immediately. This study conformed to the Declaration of Helsinki and was approved by the Ethics Committee of the Second Affiliated Hospital of Qiqihar Medical University, and all patients signed an informed consent form.

### Inclusion and exclusion criteria

Inclusion criteria: 1. onset within 12 h and persistent chest pain for more than 30 min, with no effect of sublingual nitroglycerine; 2. ST-segment elevation in at least two adjacent ECG leads (including ST-segment elevation of no less than 0.1 mV in limb leads and ST-segment elevation of no less than 0.2 mV in thoracic leads) or block in newly developed left bundle branch conduction; and 3. creatine kinase values more than two times higher than normal and positive for troponin T or I.

Exclusion criteria: 1. patients with unstable haemodynamics, including progressive hypotension, cardiogenic shock and heart failure; 2. patients with previous myocardial infarction; 3. intravenous drug thrombolysis; 4. STEMI combined with other diseases, such as valvular heart disease, dilated cardiomyopathy and severe arrhythmia; and 5. poor image quality of two-dimensional ultrasonography or contrast echocardiography.

### Apparatus

A Philips IE Elite ultrasound diagnostic instrument with an S5-1 probe at 2.5 to 3.5 MHz was used in this study, and the data analysis was completed using offline Q-Lab 9.0 software. The ultrasound contrast agent used was SonoVue (Bracco, Italy).

### Primary outcome measures

The main outcome measures of this study included the wall motion score index (WMSI), the left ventricular ejection fraction (LVEF), the A value, β value and A × β value of the MCE parameters and the effectiveness of the MCE parameters in diagnosing myocardial microcirculatory perfusion disorders in each group of patients.

### IMR measurement method

The IMR measurements were performed on the infarcted artery in the STEMI patients using a 0.014-inch guidewire with a temperature and pressure receptor at the head. The guidewire was placed two-thirds of the way away from the lesioned vessel, and then the guiding catheter was placed in the position of the coronary ostium. The pressure at the head of the guidewire was corrected to equalise with the pressure on the side of the guiding catheter. After the mean conduction time at rest was obtained by a thermodilution technique, adenosine was injected intravenously at 140 ug/(kg · min) into the body to make the coronary artery hyperaemic to the maximum state, and the mean conduction time at the maximum hyperaemia (TmnHyp) was obtained. The mean pressure in the distal and proximal coronary arteries of the stenosis was measured on the same pressure line, i.e. the Pd and Pa values were measured. Finally, the value of the IMR was calculated with the formula IMR = Pd × TmnHyp [5]. In this study, all subjects were divided into three groups using IMR values = 25 and 40 U as cut-off values. Here, IMR < 25 U was defined as the normal microcirculation group (Group 1); 25 U ≤ IMR ≤ 40 U was defined as the mildly abnormal microcirculation group (Group 2), and IMR > 40 U was defined as the severely abnormal microcirculation group, referring to previous studies (Group 3) [6, 7].

### Two-dimensional echocardiography

All patients underwent routine echocardiography one week after PCI treatment. Here, ECG monitoring was connected before the examination, and three cardiac cycle dynamic images were stored. The LVEF was measured by the biplane Simpson method. The motion of the ventricular wall segment was observed; it was scored by the 16-segment model, in which one point indicated that the motion was in a normal state, two points indicated that the motion was weakened, three points indicated that there was no motion, and four points indicated that the myocardium had paradoxical motion. The final score of the myocardium segments was obtained, and the sum of the WMS was divided by the number of ventricular wall segments to obtain the WMSI.

### MCE examination and analysis

The MCE was performed one week after PCI treatment using ultrasound contrast agent SonoVue (Bracco, Italy) in a myocardial contrast mode at a dose of 25 mg injected with 5 ml of 0.9% saline and shaken to obtain a microbubble suspension. At rest, with the patients in the left lateral decubitus position, 2.5 ml of SonoVue was withdrawn and injected intravenously into the patients over two minutes at a constant and slow rate, followed by 5 ml of saline at the same rate. The above steps could be repeated as needed. Images of the first 5 cardiac cycles before Flash and 15 cardiac cycles after Flash (MI = 1.0 at Flash and MI = 0.2 at contrast imaging) were acquired after the left ventricular myocardium was developed and stabilised. The acquisition views mainly contained apical long-axis views of left ventricular, apical two-chamber views and apical four-chamber views. After completing the examination, the images were quantitatively analysed offline with the Philips Qlab software. A region of interest (ROI), i.e. the myocardial perfusion defect area selected based on the observation of real-time myocardial contrast and the situation of the perfusion contrast (9 mm × 5 mm) was delineated in the myocardial region of the cardiac lesion, and a time-intensity curve was plotted by applying wash-in fit curve Y(t) = A × (1 – exp – βt) + C to derive quantitative results. The A value (dB) referred to the situation of the peak intensity of the contrast agent, representing the myocardium blood volume. The β value (/s) referred to the blood flow velocity of refilling after microbubble destruction, representing the blood flow velocity of the regional myocardium. The A × β value (dB/s) represented the myocardium blood flow (MBF) [8].

### Statistical methods

In this study, the SPSS 21.0 statistical software was used for the data processing. The measurement data were expressed as the mean ± standard deviation (${\overline{\text{X}}} \pm {\text{s}}$), and the enumeration data were expressed as percentages (%). A one-way analysis of variance was used to compare multiple groups that obeyed the normal distribution, with a post hoc test as LSD. A non-parametric test was used to compare multiple groups that did not obey the normal distribution. The A value, β value and A × β value were analysed with receiver operating characteristic (ROC) curves to obtain the accuracy, sensitivity and specificity for the diagnosis of myocardial microcirculatory perfusion disorders and to obtain the cut-off values of each parameter. Spearman’s correlation analysis was used for the correlation between the IMR and the A value, β value and A × β value. The results of the routine ultrasound and contrast echocardiography were analysed by two independent cardiac sonographers, and the intraclass correlation coefficient (ICC) was applied to assess the intra-operator repeatability. The enumeration data were analysed by a chi-square test. A *P*-value   0.05 was considered to indicate a statistically significant difference.

## Results

### General information

A total of 56 patients with acute STEMI were included in this study. The patients were divided into three groups according to their IMR values. There was no significant difference in gender, age, smoking history, diabetes history, hypertension history or hyperlipidaemia history among the three groups, as shown in Table 1.

**Table 1:** The comparison of the clinical information and echocardiographic findings among the three groups

Index	Total (n = 56)	Group 1 (n = 20)	Group 2 (n = 18)	Group 3 (n = 18)	*P* Value
Male	31	11	5	10	0.061
Age	59.77 ± 10.30	56.02 ± 7.67	62.57 ± 11.82	59.77 ± 10.65	0.891
Smoking history	29	9	9	11	0.921
Diabetes history	28	9	11	8	0.789
Hypertension history	28	12	7	9	0.618
Hyperlipidemia history	25	8	7	10	0.513
LVEF (%)		61.24 ± 6.25	60.22 ± 6.15	61.94 ± 4.34	
WMSI		1.22 ± 0.17	1.27 ± 0.17	1.55 ± 0.27^*#^	

*LVEF* left ventricular ejection fraction, *WMSI* ventricular wall motion score index

### Comparison of echocardiographic findings among the three groups

A total of 896 myocardial segments were identified in the 56 patients, of which 698, 124, 67 and 7 had normal, diminished, no and paradoxical motion segments, respectively. Moreover, 36 myocardial segments in Group 1, 54 myocardial segments in Group 2 and 88 myocardial segments in Group 3 showed abnormal motion. The WMSI values were calculated for each group.

There were statistically significant differences in the WMSI values between Group 3 and Groups 1 and 2 (1.49 ± 0.26 vs 1.24 ± 0.20, 1.49 ± 0.26 vs 1.27 ± 0.17; *P* < 0.05), but there was no statistically significant difference in the WMSI values between Group 1 and Group 2 (1.24 ± 0.20 vs 1.27 ± 0.17; *P* > 0.05). Meanwhile, there was no statistically significant difference in the LVEF among the three groups. See Table 1.

### Comparison of MCE results among the three groups

**Fig. 1:**
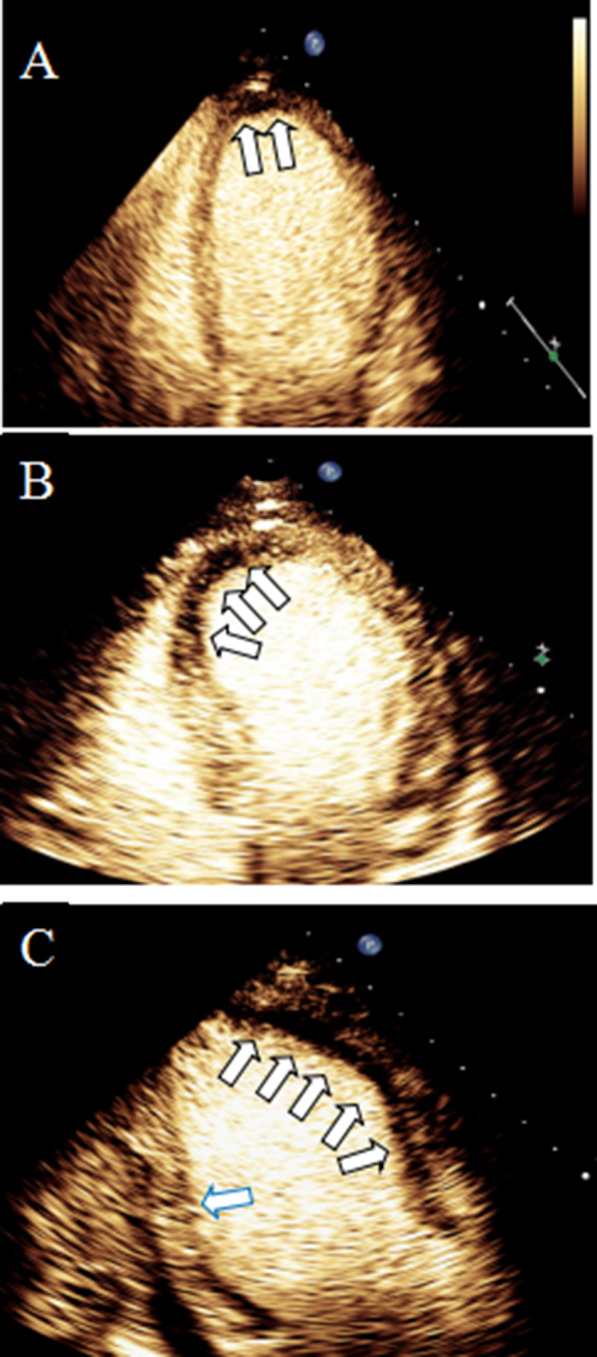
MCE images of patients in the three groups. **A** in Group 1, the perfusion of the interventricular septum and apical segment of the left ventricular lateral wall was not uniform, and a strip filling defect area was observed. **B** in Group 2, the perfusion of the interventricular septum and apical segment of the left ventricular lateral wall was significantly reduced, and a large patchy filling defect area was observed. **C** in Group 3, the perfusion of the left ventricular lateral wall was poor, and a large patchy non-perfusion area was observed. The black arrows show areas of poor myocardial perfusion, and the blue arrows show areas of good myocardial perfusion

The diseased myocardium of the patients in all three groups showed different degrees of filling defects. In Group 1, the perfusion of the diseased myocardium was fair, the filling was not uniform, and punctate and small patchy filling defect areas were observed (Fig. 1a). In Group 2, the perfusion of the diseased myocardium was poor, the filling was uneven, and large patchy filling defect areas were observed (Fig. 1b). In Group 3, the perfusion of the diseased myocardium was poor, and large patchy non-perfusion areas were observed (Fig. 1c).

**Fig. 2:**
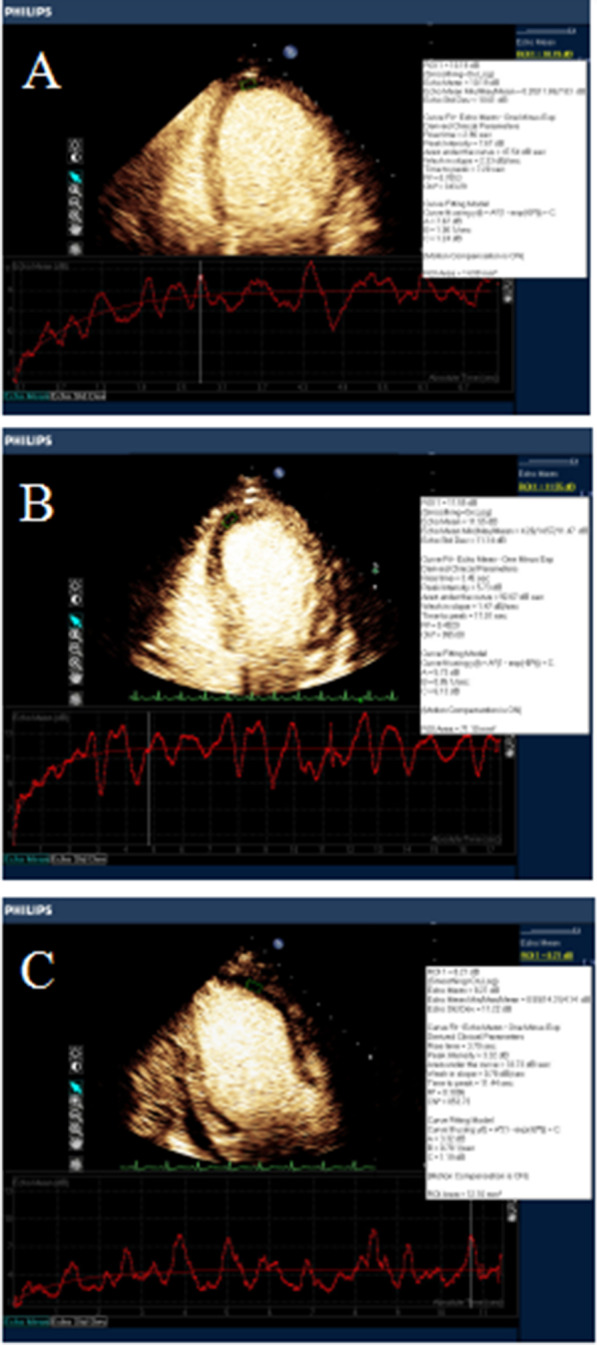
Quantitative analysis of MCE in the lesions of the three groups. **A** A value: 15.46, β value: 0.93, A × β value: 14.37 in Group 1 patients. **B** A value: 12.14, β value: 0.69, A × β value: 8.37 in Group 2 patients. **C** A value: 3.68, β value: 0.68, A × β value: 2.50 in Group 3 patients

The TIC curve of the diseased myocardium in the 56 patients was traced. In Group 1, the rising branch of the curve was steeper and straighter, with significant peak enhancement and a faster time to peak (Fig. 2a). In Group 2, the slope of the rising branch of the curve was reduced, with lower peak enhancement and a slightly longer time to peak (Fig. 2b). In Group 3, the rising branch of the curve was gentle, with significantly lower peak enhancement and a longer time to peak (Fig. 2c).

Comparison of MCE parameters among the three groups: the A value of Group 2 and Group 3 was lower than that of Group 1, and the A value of Group 3 was lower than that of Group 2 (*P* < 0.05). The β value of Group 2 and Group 3 was lower than that of Group 1, and the β value of Group 3 was lower than that of Group 2 (*P* < 0.05). The A × β value of each group decreased with the increase of the IMR value (*P* < 0.05). See Table 2.

**Table 2:** The comparison of IMR and MCE parameters among the three groups (${\overline{\text{x}}} \pm {\text{s}}$)

Index	Group 1 (n = 20)	Group 2 (n = 18)	Group 3 (n = 18)
A value (dB)	7.56 ± 2.35	6.52 ± 3.01^*^	4.61 ± 2.08^*#^
β value (/s)	1.44 ± 0.45	1.21 ± 0.58^*^	0.84 ± 0.39^*#^
A × β value (dB/s)	11.72 ± 7.79	8.59 ± 6.33^*^	4.54 ± 3.88^*#^
IMR (U)	12.77 ± 4.35	31.01 ± 5.23	75.28 ± 23.83

*IMR* microcirculation resistance index, *MCE* myocardial contrast echocardiography

*Compared with Group 1, there were statistically significant differences, *p* < 0.05

^#^Compared with Group 2, there were statistically significant differences, *p* < 0.05

### Correlation analysis

**Fig. 3:**
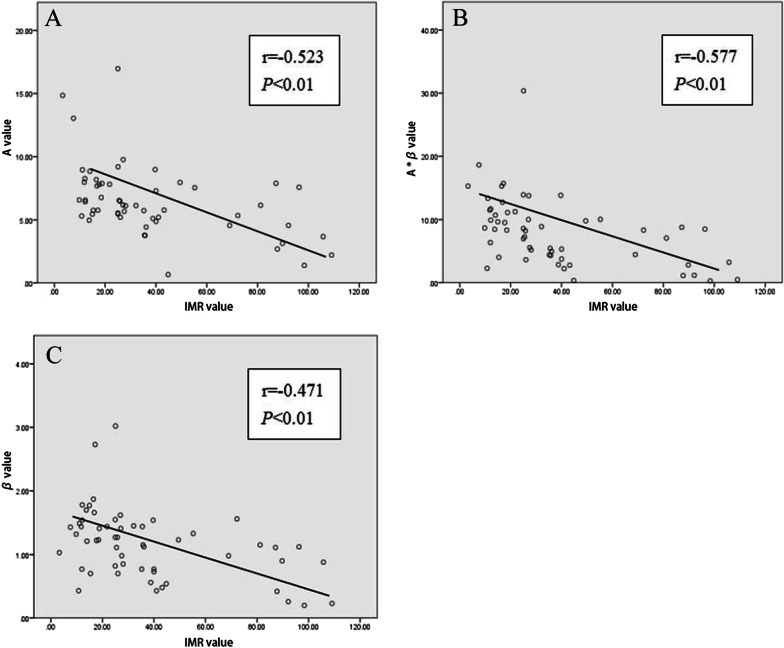
Scatter plot of the correlation analysis between the quantitative parameters of MCE and IMR. **A** The relationship between the A value and IMR value, r = −0.523. **B** The relationship between the β value and IMR value, r = –0.471. **C** The relationship between the A × β value and IMR value, r = –0.577

The results of Spearman’s correlation analysis showed that all quantitative parameters of MCE were negatively correlated with the IMR values, including the A value (r =  − 0.523, *P* < 0.01), β value (r =  − 0.471, *P* < 0.01) and A × β value (r =  − 0.577, *P* < 0.01) (see Fig. 3).

### Diagnostic value of MCE for myocardial reperfusion

**Fig. 4:**
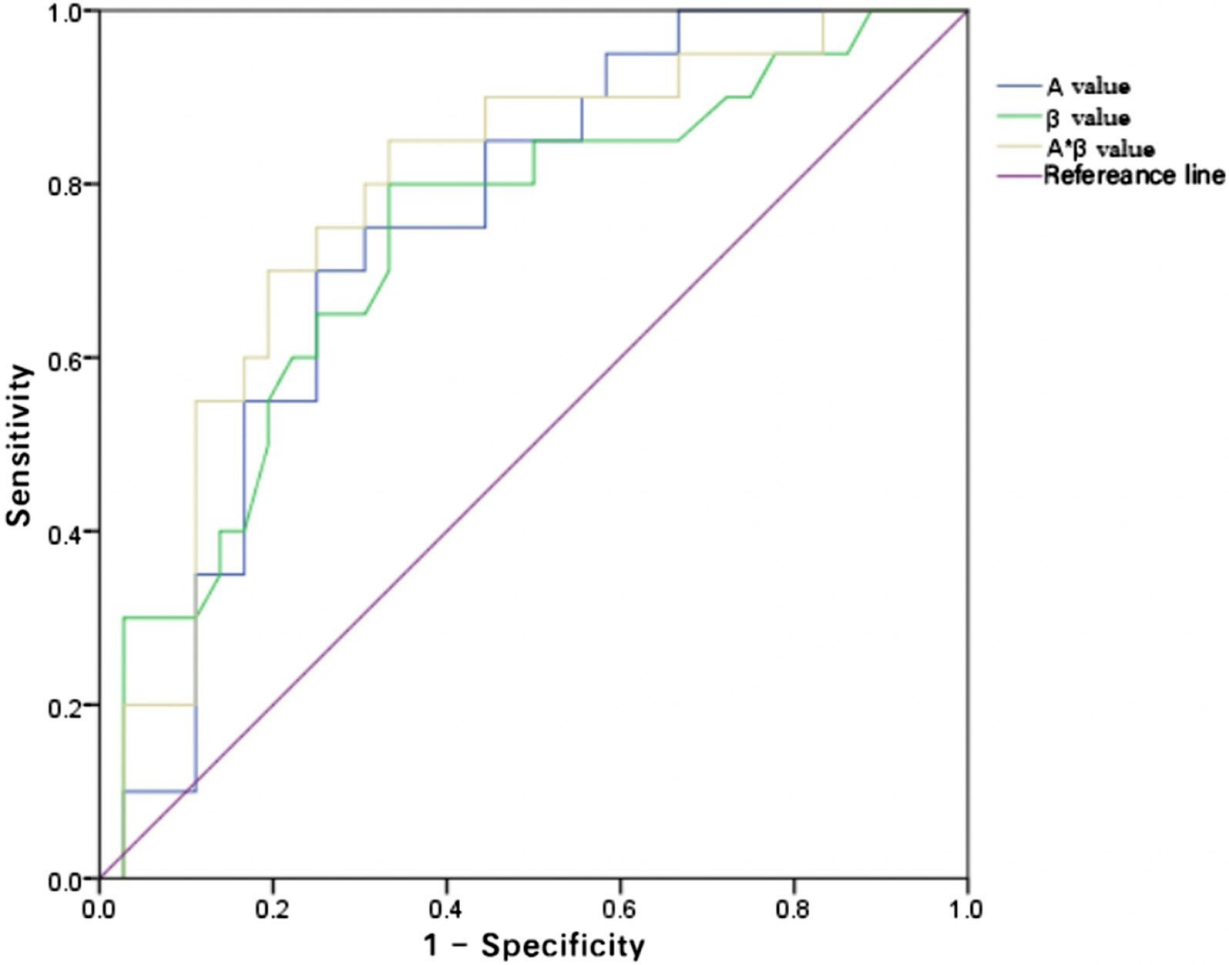
The ROC curve of MCE in the diagnosis of myocardial reperfusion disorder

The results of the ROC curves showed that the accuracy of the A × β value for diagnosing myocardial microcirculatory perfusion disorders was high, as shown in Fig. 4.

### Repeatability test

The results of the two-dimensional ultrasound and contrast echocardiography were analysed by two attending physicians, and the ICC of each group of data was > 0.85.

## Discussion

More than 25% of the patients with successful opening of the occlusion lesion of the coronary artery in the clinical trial Thrombolysis in Myocardial Infarction still had no or low flow, which was mainly associated with abnormal myocardial perfusion at the microcirculatory level [9]. The IMR is an important method for assessing coronary microcirculation and is significantly correlated with microcirculatory resistance. This examination technique is not affected by epicardial hemodynamic status and has the advantages of reliability and reproducibility [6]. Several previous studies have shown that the IMR can assess coronary microcirculatory damage after PCI in patients with acute myocardial infarction, thereby guiding coronary microcirculatory protection [10, 11]. However, measurement of the IMR is invasive, and it is technically demanding to achieve a stable maximum hyperaemic state during the examination to accurately measure the values. Therefore, there is an urgent clinical need for a non-invasive, non-radiological imaging method that can better reflect microcirculatory perfusion.

Myocardial contrast echocardiography is a new ultrasound technique widely used in clinical practice in recent years. Previous studies have found that the myocardial perfusion results evaluated by MCE are in good agreement with the myocardium blood flow obtained by SPECT and PET and that the method’s quantitative analysis diagnostic ability is equivalent to or even superior to that of SPECT [12]. Moreover, MCE can detect myocardial perfusion abnormalities and assess microvascular perfusion disorders. In this study, MCE was used to evaluate the myocardial microcirculatory perfusion at different microcirculatory resistances in patients with coronary heart disease, and the results showed that the A value, β value and A × β value were negatively correlated with the IMR. The patients with lower IMR values and higher values of various parameters of MCE had lower microcirculatory resistance and better myocardial perfusion. However, higher IMR values and lower MCE parameters indicated more severe myocardial microcirculation damage and worse myocardial perfusion. Therefore, myocardial microcirculatory perfusion can be evaluated by quantitative MCE indicators, such as the myocardium blood volume, myocardium blood flow velocity and MBF, which has some significance in judging the degree of impairment of microcirculatory function in patients with coronary heart disease.

In this study, the AUCs of the A value, β value and A × β value in the diagnosis of myocardial microcirculatory perfusion disorder were 0.756, 0.727 and 0.771, respectively, with sensitivities of 0.750, 0.800 and 0.850, respectively, and specificities of 0.755, 0.677 and 0.677, respectively, suggesting that the A x ß value is the most accurate measurement for assessing microcirculatory disorders among these MCE parameters. It has been shown in previous studies [13] that the quantitative parameters of MCE are more accurate and consistent with the A × β value in the evaluation of myocardial perfusion [14]. The main reasons for this are that the A value is affected by the location of the ROI and the sensitivity of software identification and that the β value is easily affected by local metabolites and collateral circulation; meanwhile, the A × β value can dynamically reflect the MBF and has diagnostic value.

This study has the following shortcomings. First, there was still some risk of bias, as this study was not a randomised controlled trial and no blind method was set. Second, this study was a single-centre clinical study; a multicentre clinical study is still needed for further investigation. Finally, the sample size included in this study was small, and further research with a larger sample size is still needed.

## Conclusions

In conclusion, the A value, β value and A × β value of MCE are correlated with the IMR value. Therefore, the application of MCE could be used to observe the myocardial perfusion after PCI in patients with acute STEMI and accurately assess the myocardial microcirculation. It is worthy of being widely popularised in clinical applications.

## Data Availability

All data generated or analyzed during this study are included in this published article.
